# RanBPM Is an Inhibitor of ERK Signaling

**DOI:** 10.1371/journal.pone.0047803

**Published:** 2012-10-31

**Authors:** Elnaz Atabakhsh, Caroline Schild-Poulter

**Affiliations:** Robarts Research Institute and Department of Biochemistry, Schulich School of Medicine and Dentistry, The University of Western Ontario, London, Ontario, Canada; University of Sherbrooke, Canada

## Abstract

Ran-binding protein M (RanBPM) is a nucleocytoplasmic protein of yet unknown function. We have previously shown that RanBPM inhibits expression of the anti-apoptotic factor Bcl-2 and promotes apoptosis induced by DNA damage. Here we show that the effects of RanBPM on Bcl-2 expression occur through a regulation of the ERK signaling pathway. Transient and stable down-regulation of RanBPM stimulated ERK phosphorylation, leading to Bcl-2 up-regulation, while re-expression of RanBPM reversed these effects. RanBPM was found to inhibit MEK and ERK activation induced by ectopic expression of active RasV12. Activation of ERK by active c-Raf was also prevented by RanBPM. Expression of RanBPM correlated with a marked decrease in the protein levels of ectopically expressed active c-Raf and also affected the expression of endogenous c-Raf. RanBPM formed a complex with both active c-Raf, consisting of the C-terminal kinase domain, and endogenous c-Raf in mammalian cells. In addition, RanBPM was found to decrease the binding of Hsp90 to c-Raf. Finally, we show that loss of RanBPM expression confers increased cell proliferation and cell migration properties to HEK293 cells. Altogether, these findings establish RanBPM as a novel inhibitor of the ERK pathway through an interaction with the c-Raf complex and a regulation of c-Raf stability, and provide evidence that RanBPM loss of expression results in constitutive activation of the ERK pathway and promotes cellular events leading to cellular transformation and tumorigenesis.

## Introduction

The ERK pathway is activated by a wide range of signals including growth factors, cytokines and external stressors. These signals trigger the activation of transmembrane receptors such as receptor tyrosine kinase (RTK) or G protein-coupled receptors which activate the Ras-Raf-MEK signaling cascade [1], [2]. Activation of Ras is mediated by adaptor proteins, including Sos (son-of-sevenless) and Grb2 (growth-factor-receptor bound 2), which mediate GDP for GTP exchange on Ras, leading to Ras activation [1], [3]. Activation of Ras at the plasma membrane leads to its association with Raf serine/threonine kinases, promoting their activation and in turn phosphorylation and activation of MEK1/2, ultimately resulting in the activation of ERK1 and ERK2 [1], [3]. ERK1 and ERK2 (commonly referred to as ERK1/2 or ERK) are over 80% identical and share many physiological functions. ERK1/2 are promiscuous kinases that have been demonstrated to act on nearly 100 cellular targets, and regulate several diverse cellular functions such as cell cycle progression, proliferation, cell adhesion, transcription, and importantly cell death and apoptosis [3], [4]. The ERK pathway is generally associated with increased cell survival and proliferation and has been shown to be constitutively activated in many tumours [4], [5]. In particular, the ERK pathway is known to inhibit apoptosis by regulating the levels and activity of many apoptotic regulators, including Bcl-2 and Bcl-X_L_ [4], [6], [7].

Ran-binding protein M (RanBPM, also called RanBP9) is a nucleocytoplasmic protein whose function is still elusive, but that has been implicated in a variety of cellular functions, including transcriptional regulation [8], [9], regulation of cell morphology [10], [11] and regulation of receptor-activated intracellular signaling pathways including those activated by MET, TrkA and TrkB [12], [13], [14], [15]. Analyses of RanBPM-deficient mice have recently shown a role for RanBPM in gametogenesis in both genders [16]. Several reports have also suggested that RanBPM functions as a regulator of apoptotic pathways through its interaction with several apoptotic regulators such as cyclin-dependent kinase CDK11p46, the p75 neurotrophin receptor (p75NTR), p73, and homeodomain interacting protein kinase-2 (HIPK-2) [17], [18], [19], [20]. Recently, we demonstrated a functional role for RanBPM in DNA-damage induced activation of the intrinsic apoptotic pathway [21]. We found that down-regulation of RanBPM inhibited the activation of apoptosis in response to ionizing radiation (IR), and consequently led to increased cell survival in both Hela and HCT116 cells. Furthermore, we showed that down-regulation of RanBPM resulted in a substantial up-regulation of Bcl-2 protein levels, suggesting that RanBPM pro-apoptotic function could result at least in part from its ability to regulate the expression anti-apoptotic factors.

In the present study we provide evidence that the RanBPM-mediated regulation of Bcl-2 is linked to its regulation of the ERK pathway. First we show that, similarly to Bcl-2, the protein levels of Bcl-X_L_ are markedly increased in RanBPM down-regulated cells and that RanBPM controls the expression of these anti-apoptotic factors both at the transcriptional and post-translational levels. Next, we demonstrate that RanBPM down-regulation results in increased ERK1/2 activation that can be reversed upon re-expression of RanBPM, and that the effect of RanBPM on Bcl-2 expression is dependent on the regulation of the ERK1/2 pathway by RanBPM. Furthermore, we provide evidence that RanBPM's control of ERK signaling occurs through a regulation of c-Raf levels/stability and that RanBPM associates with c-Raf and affects the interaction of c-Raf and Hsp90. Finally, we show that RanBPM down-regulation promotes cell proliferation and migration, cell transformation properties known to be triggered by deregulated ERK activation. Together, our findings implicate a novel role for RanBPM as an inhibitor of ERK1/2 activation through the regulation of c-Raf stability. They also suggest that loss of RanBPM function, in addition to compromising apoptosis, promotes cellular events leading to cellular transformation, and that these effects could be attributed, at least in part, through a deregulation of the ERK pathway.

## Results

### RanBPM modulates transcriptional and post-transcriptional events that regulate Bcl-2 and Bcl-X_L_ expression

**Figure 1:**
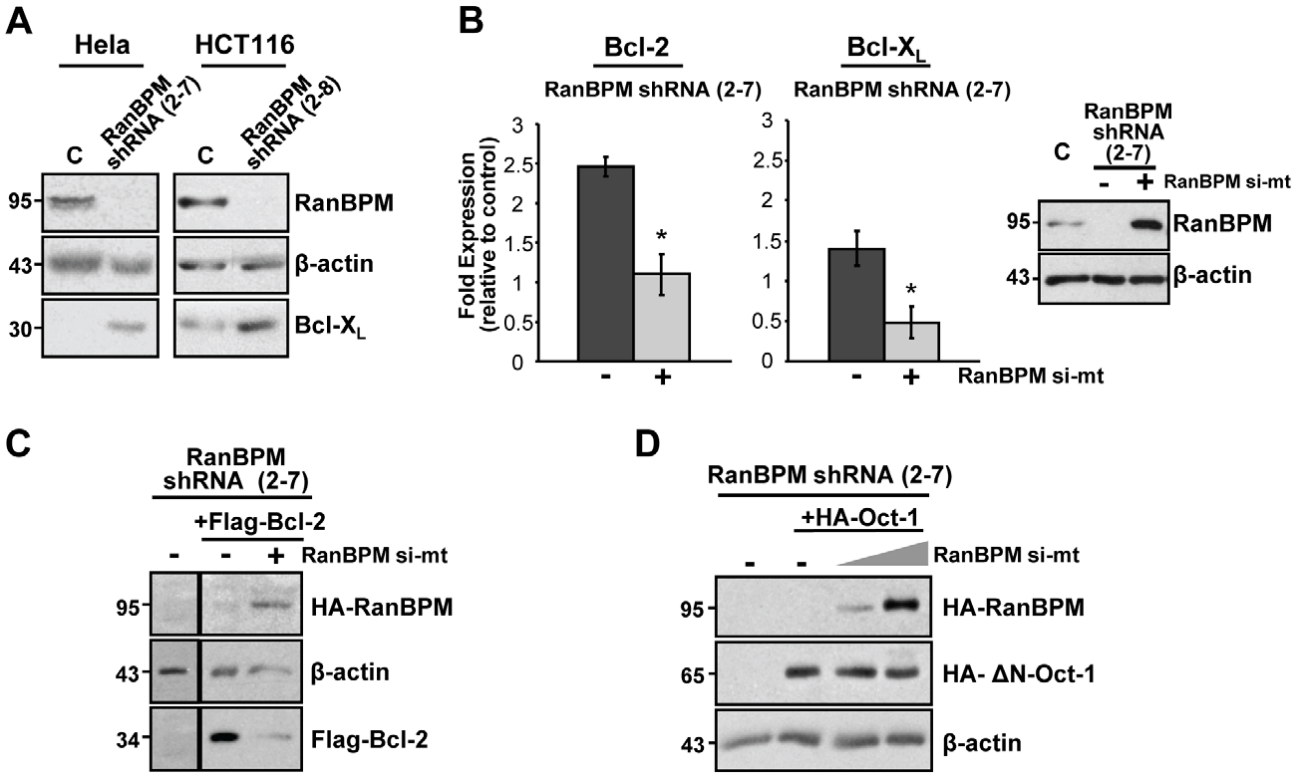
Regulation of Bcl-2 and Bcl-X_L_ expression by RanBPM. (**A**) Down-regulation of RanBPM leads to enhanced Bcl-X_L_ expression. Whole cell extracts were prepared from Hela and HCT116 control shRNA and RanBPM shRNA cells and were analyzed by western blotting. Blots were hybridized with antibodies against Bcl-X_L_, β-actin and RanBPM. (**B**) RanBPM shRNA cells exhibit enhanced Bcl-2 and Bcl-X_L_ mRNA expression. cDNA from Hela control shRNA, RanBPM shRNA, and RanBPM shRNA cells re-expressing RanBPM via transient transfection of RanBPM si-mt construct was analyzed by qRT-PCR with RNA polymerase II (Pol II), Bcl-2, and Bcl-X_L_, specific primers. Relative quantification of Bcl-2 and Bcl-X_L_ gene expression was determined using the ΔΔC(t) method with Bcl-2 and Bcl-X_L_ expression normalized to that of the controls. Bars represent values normalized to control shRNA cells. Data represents the mean of three independent experiments with error bars representing standard deviation (SD). *, *P*<0.05. *Inset*, representative western blot analysis of whole cell extracts to control for the levels of RanBPM using a RanBPM antibody and β-actin as a loading control. (**C**) RanBPM expression down-regulates Bcl-2 protein levels. Hela RanBPM shRNA cells were transfected with pCMV-3xFlag-Bcl-2. 24 h post-transfection, cells were split and were either transfected with pCMV-HA-RanBPM si-mt or empty vector. Whole cell extracts were prepared 48 h later and analyzed by western blotting. Expression of ectopic Bcl-2 was determined by hybridization with anti-Flag antibody. RanBPM expression was assessed with a RanBPM antibody, and β-actin was used as a loading control. (**D**) Control experiment to confirm the specificity of RanBPM expression on Bcl-2 protein levels. This experiment was carried out the same as in C, except that RanBPM shRNA cells were transfected with pCGN-HA-ΔN-Oct-1 instead of Flag-Bcl-2. The truncated ΔN-Oct-1 migrates at 65 kDa as opposed to full-length Oct-1 (which migrates at 90 kDa), allowing for detection of Oct-1 and RanBPM expression in cells transfected with both constructs. Blots were hybridized with anti-HA antibody to verify Oct-1 expression.

We showed previously that down-regulation of RanBPM expression leads to increased Bcl-2 protein levels in Hela and HCT116 cells [21]. We expanded these analyses to determine whether the expression of other anti-apoptotic Bcl-2 family factors such as Bcl-X_L_ and Mcl-1 was also altered in the absence of RanBPM. Analysis of whole cell extracts from control small hairpin RNA (shRNA) and RanBPM shRNA Hela and HCT116 revealed that Bcl-X_L_ protein levels were markedly elevated in RanBPM shRNA cells compared to control cells (Fig. 1A). However, we found that Mcl-1 protein levels remain unchanged in RanBPM shRNA cells (data not shown).

We carried out quantitative reverse transcriptase-PCR (qRT-PCR) analyses to determine whether RanBPM is involved in the regulation of Bcl-2 and Bcl-X_L_ gene expression. RanBPM shRNA cells showed increased mRNA levels for both Bcl-2 (2.5-fold increase) and Bcl-X_L_ (1.4- fold increase) in comparison to control shRNA cells (Fig. 1B). To verify that this increase in gene expression was specifically due to RanBPM down-regulation, we re-expressed RanBPM in RanBPM shRNA cells by transfecting a RanBPM cDNA containing a point mutation in the shRNA target sequence (RanBPM si-mt) [21]. Upon RanBPM re-expression, Bcl-2 and Bcl-X_L_ mRNA expression was reduced to levels near that of control shRNA cells (Fig. 1B), thus confirming a role for RanBPM in the transcriptional regulation of Bcl-2 and Bcl-X_L_. Previous reports have implicated RanBPM in the regulation of protein stability [17], [22], therefore we sought to determine whether RanBPM may also regulate Bcl-2 and/or Bcl-X_L_ protein levels. To this end, we expressed a Flag-Bcl-2 construct under the control of the CMV promoter in RanBPM shRNA cells and analyzed the effect of RanBPM re-expression on the Flag-Bcl-2 protein levels. Expression of RanBPM led to a significant down-regulation of Flag-Bcl-2 protein levels (Fig. 1C). To ensure that this was not due to an effect of RanBPM on the CMV promoter, we repeated this experiment using an Octamer transcription factor-1 (Oct-1) expression construct also under the control of a CMV promoter. Endogenous Oct-1 protein levels are not affected by RanBPM down-regulation (data not shown). As Oct-1 and RanBPM migrate at the same size on SDS-PAGE (about 95 kDa), a truncated form of Oct-1 (HA-ΔN-Oct-1 [23]) lacking Oct-1 N-terminus was used so that ectopically expressed HA-Oct-1 and HA-RanBPM would be detected on the same gel. Oct-1 protein levels were found unchanged upon RanBPM expression (Fig. 1D), indicating that RanBPM does not modulate the activity of the CMV promoter, thus confirming a regulation of Bcl-2 by RanBPM through a post-transcriptional or post-translational mechanism.

### RanBPM inhibits ERK1/2 activation

**Figure 2:**
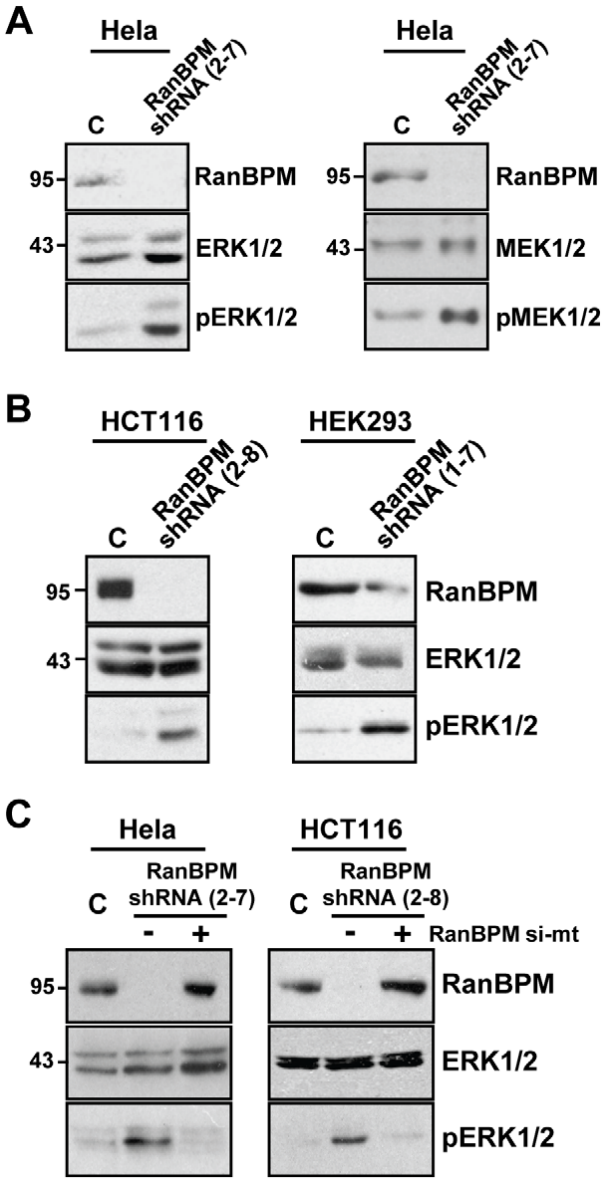
RanBPM is a negative regulator of ERK1/2 activation. (**A**) Enhanced MEK1/2 and ERK1/2 phosphorylation in the absence of RanBPM. Whole cell extracts were prepared from Hela control shRNA and RanBPM shRNA cells and analyzed by western blotting. Activation of ERK1/2 was determined by hybridization with phospho-ERK1/2 antibody. Expression of RanBPM in control shRNA cells was verified using a RanBPM antibody, and total ERK1/2 was used as a loading control. (**B**) HCT116 control shRNA and RanBPM shRNA cells were serum-starved for 18 h in 0.1% FBS. HEK293 control shRNA and RanBPM shRNA cells were incubated in serum-free media for 24 h. Whole cell extract were analyzed as described in A. (**C**) Control and RanBPM shRNA Hela and HCT116 cells were either left untransfected, or were transfected with empty vector or RanBPM si-mt. 24 h post-transfection, HCT116 cells were serum-starved in 0.1%FBS, and extracts were prepared 18 h later. Western blot analysis was carried out the same as in A.

Our findings of a regulation by RanBPM of Bcl-2 and Bcl-X_L_ expression through mechanisms involving transcriptional and post-translational regulations suggested that RanBPM could regulate signaling pathway(s) that control the expression of both factors. One of the main pathways that regulate Bcl-2 (and Bcl-X_L_) both transcriptionally and post-transcriptionally is the ERK pathway [4], [5]. RanBPM was previously shown to participate in ERK1/2 signaling, but the effects of RanBPM on this pathway remain controversial [12], [24], [25]. Thus, we looked at a direct effect of RanBPM down-regulation on ERK activation by comparing ERK1/2 and MEK1/2 phosphorylation in extracts from Hela control shRNA and RanBPM shRNA cells. Both MEK1/2 and ERK1/2 phosphorylation was significantly up-regulated in Hela RanBPM shRNA cells compared to control cells (Fig. 2A). To verify that this enhanced ERK1/2 activation was not specific to Hela cells, we prepared extracts from serum-deprived HCT116 control and RanBPM shRNA cells. We observed a similar up-regulation in ERK1/2 phosphorylation in RanBPM shRNA HCT116 cells compared to control cells (Fig. 2B). We also generated a third stable cell line by expressing either the control shRNA or RanBPM shRNA in HEK293 cells, which are immortalized but not transformed [26]. Similarly to the effect observed in HCT116 cells, serum starvation led to enhanced ERK1/2 phosphorylation in RanBPM-down-regulated HEK293 cells compared to control shRNA cells (Fig. 2B). Finally, we verified that the increased ERK1/2 activation was due to a lack of RanBPM expression, as re-expression of RanBPM in both RanBPM shRNA Hela and HCT116 cells led to a marked down-regulation of ERK1/2 phosphorylation to levels near that of control cells (Fig. 2C). Altogether, these analyses suggested that RanBPM inhibits ERK phosphorylation and that down-regulation of RanBPM leads to a constitutive ERK activation.

### Inhibition of ERK1/2 signaling down-regulates Bcl-2 protein levels in RanBPM shRNA cells

**Figure 3:**
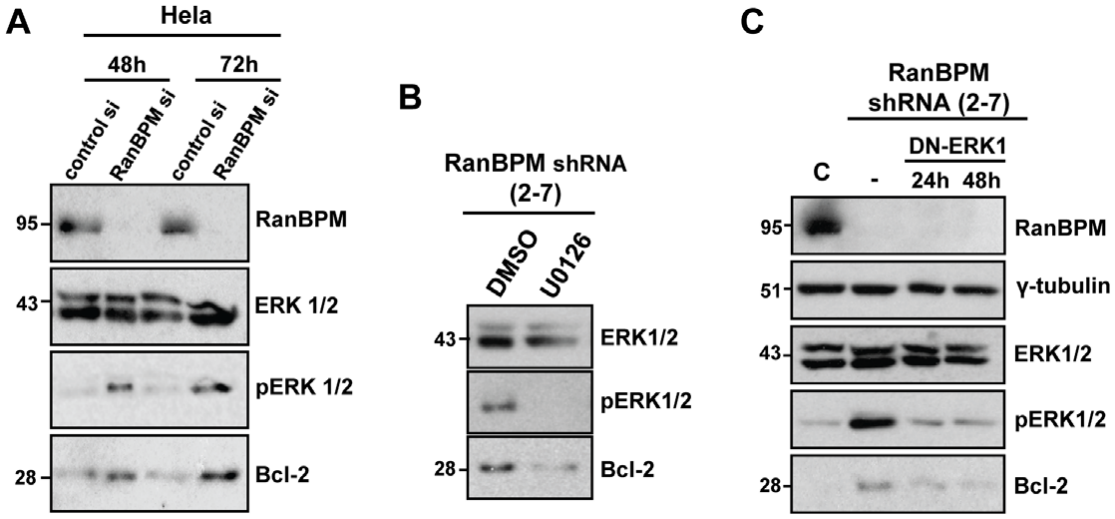
Regulation of Bcl-2 expression by RanBPM occurs through ERK1/2. (**A**) Transient down-regulation of RanBPM increases ERK1/2 phosphorylation and Bcl-2 protein levels. Hela cells were transfected with control siRNA or RanBPM siRNA. Whole cell extracts were prepared at the indicated timepoints and analyzed by western blotting with the indicated antibodies. (**B**) RanBPM shRNA Hela cells were treated with the MEK1/2 inhibitor U0126 or DMSO for 24 h. Whole cell extracts were prepared and analyzed by western blot as in A. (**C**) Hela control and RanBPM shRNA cells were either left untransfected, or were transfected with DN-ERK1 and whole cell extracts were collected 24 h and 48 h post-transfection. Western blot analysis was performed as in A with γ-tubulin used as a loading control.

To further confirm that the enhanced ERK1/2 activation was due to a decrease of RanBPM expression, we performed transient small interfering RNA (siRNA) knockdown experiments in Hela cells (Fig. 3A). Transient down-regulation of RanBPM correlated with a marked increase in ERK1/2 phosphorylation, and this correlated with an increase in Bcl-2 protein levels, suggesting a direct link between RanBPM expression, ERK pathway activation, and Bcl-2 up-regulation.

Since activation of the ERK pathway has been shown to enhance Bcl-2 expression [4], [5], [27], we assessed whether the increased ERK1/2 phosphorylation in RanBPM shRNA cells was responsible for the elevated Bcl-2 protein levels observed in these cells. Treatment of RanBPM shRNA cells with the MEK1/2 inhibitor U0126 completely abolished ERK1/2 phosphorylation, and coincided with a marked down-regulation of Bcl-2 protein expression (Fig. 3B). U0126 however is not entirely specific to the ERK1/2 pathway, but can also inhibit MEK5 and thus the whole ERK5 pathway [28]. Therefore we repeated this experiment using a dominant negative ERK1 cDNA construct (DN-ERK1) [29]. Expression of DN-ERK1 led to a down-regulation in ERK1/2 phosphorylation and correlated with a significant down-regulation in Bcl-2 protein levels (Fig. 3C). These findings support the notion that the enhanced ERK1/2 activation resulting from RanBPM down-regulation promotes the up-regulation of Bcl-2 expression.

### RanBPM targets the ERK1/2 signaling pathway downstream of Ras

**Figure 4:**
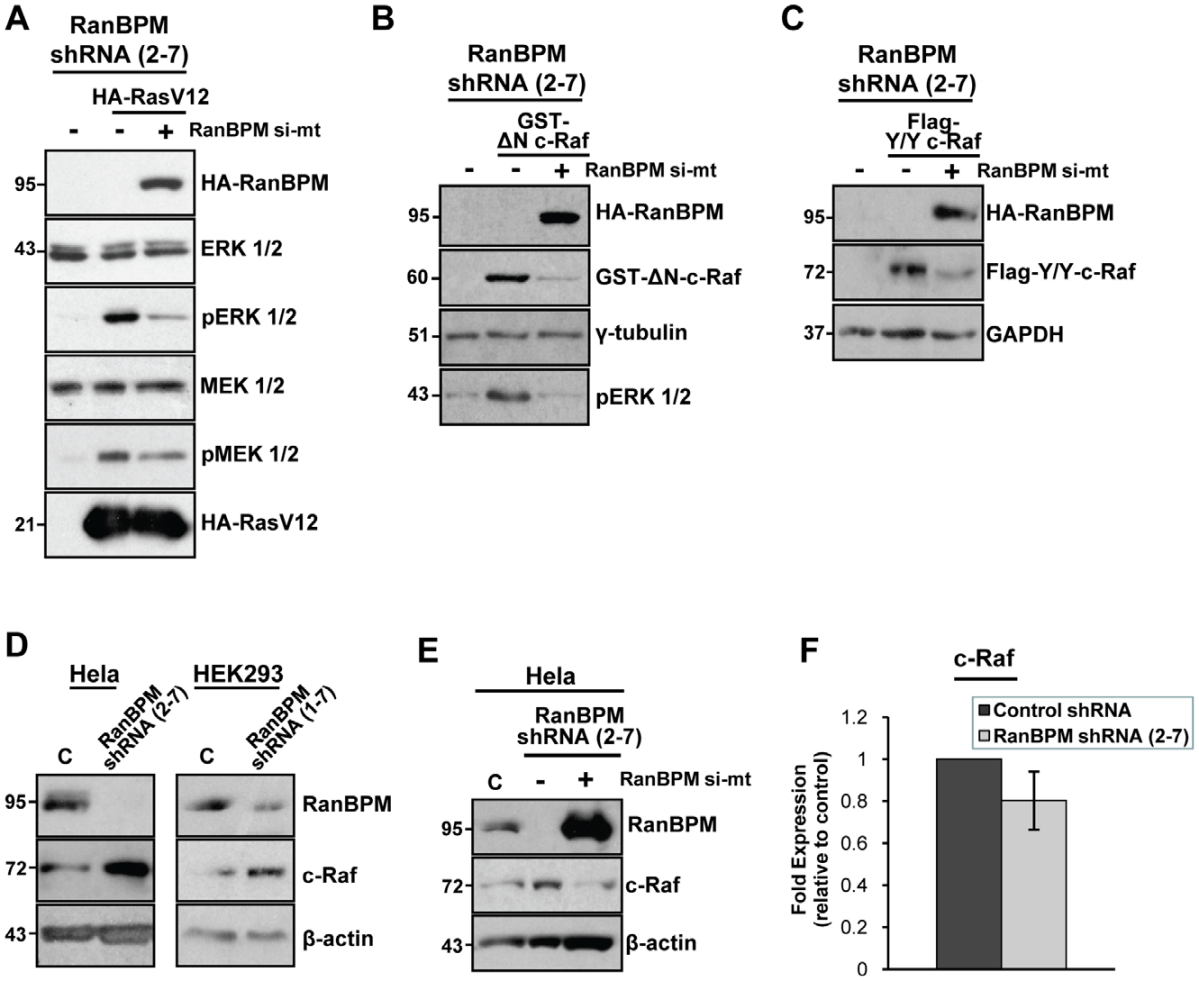
RanBPM inhibits ERK1/2 activation through regulation of c-Raf. (**A**) RanBPM regulates ERK1/2 signaling downstream of Ras. RanBPM shRNA Hela cells were left untransfected, or were transfected with either constitutively active RasV12 and RanBPM si-mt or RasV12 and empty pCMV vector. 24 h post-transfection, whole cell extracts were prepared and analyzed by western blotting. MEK1/2 and ERK1/2 activation was assessed by hybridization with phospho-MEK1/2 and phospho-ERK1/2 antibodies respectively and total MEK1/2 and total ERK1/2 levels were assessed using MEK1/2 and ERK1/2 antibodies. Expression of RasV12 and RanBPM was determined with an HA antibody. (**B**) RanBPM expression down-regulates c-Raf protein levels. RanBPM shRNA Hela cells were left untransfected, or were transfected with either constitutively active c-Raf (pEBG-GST-ΔN-c-Raf) and empty pCMV vector, or GST-ΔN-c-Raf and RanBPM si-mt. 48 h post-transfection, whole cell extracts were prepared and analyzed by western blotting. c-Raf expression was determined using a GST antibody, and ERK1/2 activation was assessed using a phospho-ERK1/2 antibody. RanBPM expression was verified using an HA antibody, and γ-tubulin was used as a loading control. (**C**) RanBPM shRNA Hela cells were either left untransfected or were transfected with constitutively active c-Raf (pCMV-Flag-Y/Y-c-Raf) and 48 h post-transfection, whole cell extracts were prepared as in B. Expression of c-Raf was assessed using a Flag antibody, and RanBPM expression was determined using an HA antibody. GAPDH was used as a loading control. (**D**) Whole cell extracts were prepared from Hela and HEK293 control shRNA and RanBPM shRNA cells and endogenous protein levels were analyzed by western blotting with c-Raf and RanBPM antibodies, with β-actin used as a loading control. (**E**) Control and RanBPM shRNA Hela cells were either left untransfected, or were transfected with empty vector or RanBPM si-mt. 48 h post-transfection, whole cell extracts were prepared and analyzed as in D. (**F**) RanBPM down-regulation does not affect c-Raf mRNA levels. cDNA from Hela control and RanBPM shRNA cells was analyzed by qRT-PCR using specific primers for GAPDH and c-Raf. Gene expression was quantified using the ΔΔC(t), with c-Raf expression normalized to GAPDH. Expression in RanBPM shRNA cells was calculated relative to that of control shRNA cells (set to an arbitrary value of 1). Data represents the mean of nine independent experiments, with error bars indicating standard error (SE).

We investigated the ability of RanBPM to regulate upstream events in the ERK signaling cascade. Both active forms of Ras and c-Raf have been shown to bypass the upstream components of the pathway for ERK activation [1], [4], [30]. As a first step, we assessed whether RanBPM acts upstream or downstream of Ras to inhibit ERK1/2 activation. We expressed a constitutively active H-Ras construct (RasV12) in Hela RanBPM shRNA cells, either in the presence or absence of ectopically expressed RanBPM, and analyzed the effect of RasV12 and RanBPM expression on the levels of ERK1/2 phosphorylation. As expected, while RasV12 expression resulted in increased ERK phosphorylation, this effect was inhibited by RanBPM expression (Fig. 4A). Interestingly, MEK-induced phosphorylation by RasV12 was also reduced upon RanBPM expression. This suggested that RanBPM is able to inhibit MEK and ERK activation by Ras and thus functions to regulate signaling events between Ras and MEK.

### RanBPM forms a complex with c-Raf and inhibits c-Raf expression

**Figure 5:**
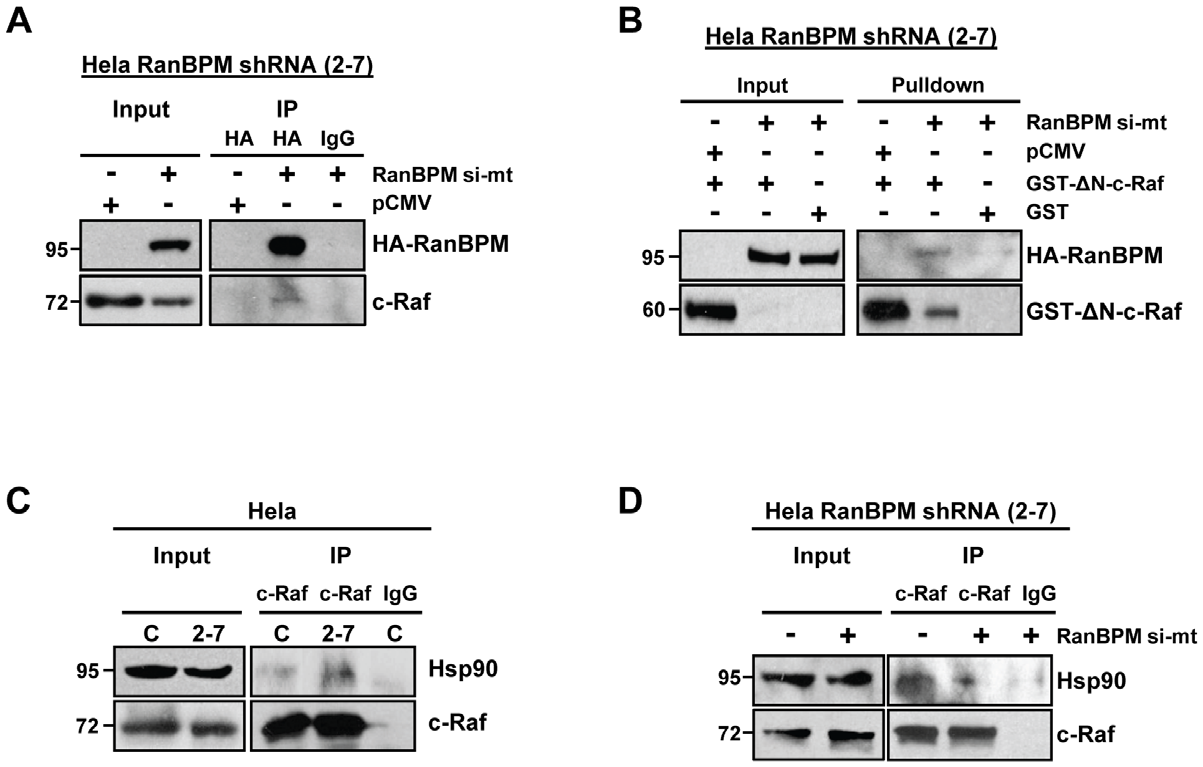
RanBPM interacts with c-Raf and reduces c-Raf-Hsp90 association. (**A**) Co-immunoprecipitation of RanBPM and c-Raf. RanBPM shRNA Hela cells were transfected with empty vector or RanBPM si-mt, and 48 post-transfection whole cell extracts were incubated with either an HA antibody or mouse IgG control. Presence of c-Raf in immunoprecipitates was determined using a c-Raf antibody and RanBPM expression was verified using HA, compared to 5% input extract. (**B**) RanBPM shRNA Hela cells were transfected with GST-ΔN-c-Raf and either pCMV empty vector or RanBPM si-mt, or with RanBPM si-mt and GST empty vector, and whole cell extracts were prepared 48 h post-transfection. Activated c-Raf was pulled down using glutathione-agarose beads, the presence of RanBPM was assessed using an HA antibody, and c-Raf expression was determined using a GST antibody, compared to 5% input extract. (**C**) Co-imunoprecipitation of Hsp90 with c-Raf. Extracts from Hela control (C) and RanBPM shRNA cells (2–7) were immunoprecipitated with c-Raf or mouse IgG control antibodies. Equal amounts of immunoprecipitated c-Raf from control and RanBPM shRNA cells were run on SDS-PAGE and analyzed by western blot with Hsp90 and c-Raf antibodies. Inputs represent 5% of the total protein used for immunoprecipitation. (**D**) Hela RanBPM shRNA cells were transfected with empty vector (-) or RanBPM si-mt, and whole cell extracts prepared 48 post-transfection were immunoprecipitated and analyzed as in C.

Using a similar experimental scheme, we next investigated whether RanBPM expression could inhibit MEK and ERK activation by active c-Raf. Co-expression of RanBPM with a constitutively active c-Raf construct (GST-ΔN-c-Raf, containing c-Raf aa 325–648 [31]) in Hela RanBPM shRNA cells had an inhibitory effect on c-Raf-induced ERK activation (Fig. 4B). Intriguingly, we found that expression of RanBPM consistently led to a pronounced decrease in GST-ΔN-c-Raf protein levels (Fig. 4B, see also Fig. 5B). Since the GST-ΔN-c-Raf construct is under a EF-1alpha promoter that could potentially be affected by RanBPM expression, this experiment was repeated with another constitutively active c-Raf construct (c-Raf Y340D/Y341D) that is expressed from a CMV promoter, which transcriptional activity is not affected by RanBPM (see Fig. 1D). We obtained a similar down-regulation of c-Raf Y340D/Y341D upon RanBPM expression (Fig. 4C), suggesting that RanBPM functions to down-regulate c-Raf protein levels.

To confirm that RanBPM affects c-Raf expression, we analyzed endogenous c-Raf protein levels in Hela and HEK293 control and RanBPM shRNA cells (Fig. 4D). Indeed, RanBPM shRNA cells exhibited elevated c-Raf protein levels, and this effect was specifically due to RanBPM down-regulation, as restoration of RanBPM expression in RanBPM shRNA cells led to a decrease in c-Raf protein levels (Fig. 4E). To determine whether up-regulation of endogenous c-Raf protein levels may also be attributed to changes in c-Raf gene expression, we performed qRT-PCR analyses to compare c-Raf gene expression in Hela control and RanBPM shRNA cells. Surprisingly, RanBPM down-regulation resulted in a slight decrease in c-Raf mRNA expression, although this difference was not found to be statistically significant, suggesting that RanBPM does not affect c-Raf expression at the transcriptional level (Fig. 4F). Together these findings indicate that RanBPM functions to regulate ERK1/2 signaling by modulating c-Raf protein levels.

To start investigating how RanBPM promotes c-Raf down-regulation, we first looked into a possible association of RanBPM with the c-Raf complex. A previous study reported the interaction of c-Raf kinase domain with RanBPM in a yeast two-hybrid analysis, but their interaction was not confirmed in mammalian cells [24]. Endogenous c-Raf was found to co-immunoprecipitate with HA-RanBPM re-expressed in RanBPM shRNA cells, suggesting that the two proteins form a complex (Fig. 5A). In addition, we determined that GST-ΔN-c-Raf, the levels of which were found to be markedly affected by RanBPM (Figs. 4B and 5B), was able to interact with RanBPM (Fig. 5B). Altogether, these results suggest that RanBPM associates with c-Raf and that this interaction relies on the c-Raf C-terminal kinase domain.

### RanBPM disrupts Hsp90-c-Raf association

Rafs are Hsp90 client proteins, and the binding of Hsp90 to c-Raf is required for proper folding and is essential for c-Raf protein stability [32], [33]. To begin to characterize the mechanism by which RanBPM may regulate c-Raf, we assessed whether RanBPM expression affected the association of Hsp90 with c-Raf. Co-immunoprecipitation of c-Raf from Hela control and RanBPM shRNA cell extracts revealed an increased amount of Hsp90 co-immunoprecipitating with c-Raf in RanBPM-depleted cells (Fig. 5C). In addition, re-expression of RanBPM reversed this effect, substantiating an inhibitory effect of RanBPM on the association of Hsp90 with c-Raf (Fig. 5D). Altogether, these findings indicate that RanBPM may function to destabilize the c-Raf protein by inhibiting the interaction of c-Raf and Hsp90.

### Inhibition of RanBPM expression promotes cellular transformation

**Figure 6:**
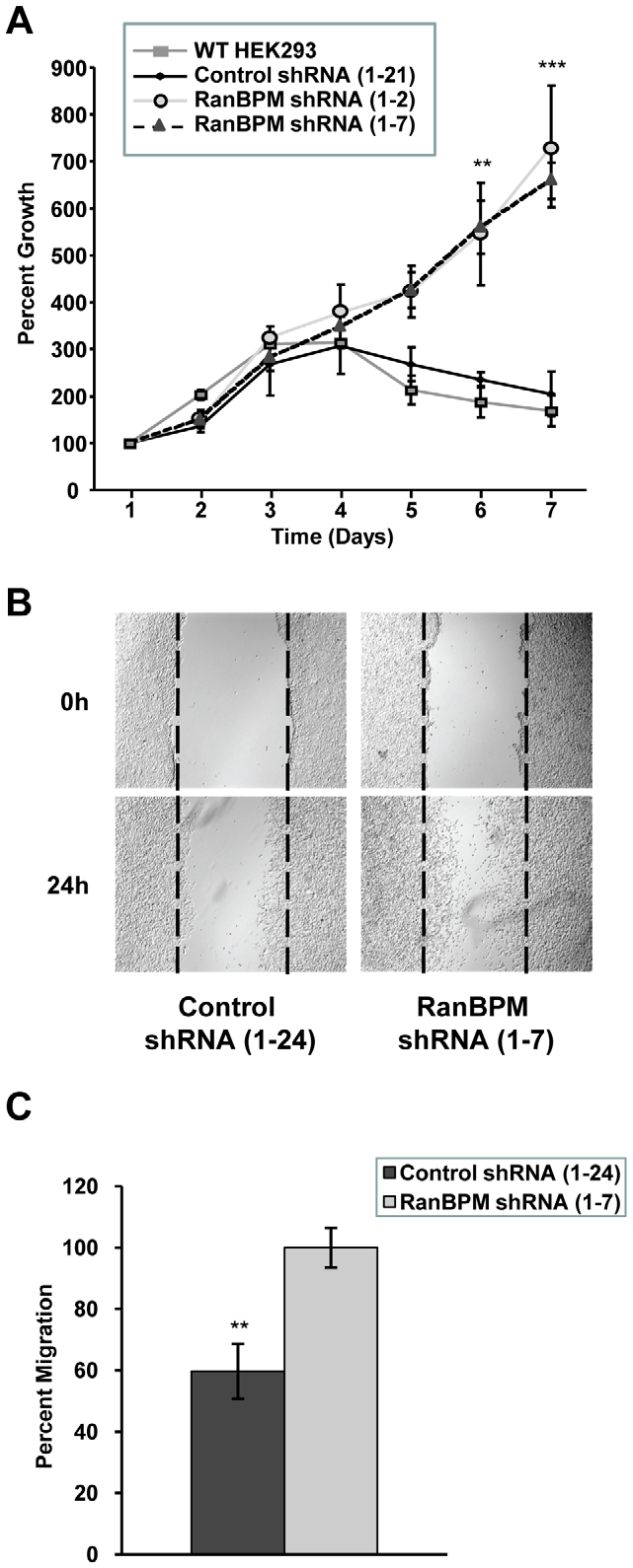
Down-regulation of RanBPM expression enhances cellular transformation. (**A**) RanBPM shRNA cells exhibit increased cell growth. Growth rates for HEK293 wild-type (WT), control shRNA and RanBPM shRNA cells were assessed for 7 days. Data represents mean percent growth for four independent experiments, with error bars indicating SE. ** indicates *P*<0.01 and *** indicates *P*<0.001. (**B**) Confluent monolayers of control and RanBPM shRNA HEK293 cells were cultured in the presence of 2 mM hydroxyurea for 24 h, scratched using a sterile pipette tip and wound healing was assessed at the indicated time points using a microscope at 4× magnification. Images from a representative experiment are shown. (**C**) Percent wound closure was calculated for control and RanBPM shRNA HEK293 cells. Data represents the mean of four independent experiments with error bars representing SE, and ** indicating *P*<0.01.

Activating mutations in K-Ras and N-Ras leading to the constitutive activation of the ERK pathway are among the most frequent oncogenic events in human cancers [5], [34]. Our observation that RanBPM down-regulation promotes ERK activation suggested that loss of RanBPM function, in addition to compromising apoptosis, could promote cellular events leading to cellular transformation. We first analyzed the effect of RanBPM down-regulation on cell growth in HEK293 cells. We evaluated the growth rate of both RanBPM and control shRNA HEK293 cells upon serum withdrawal. For both HEK293 (WT) and the clonal derivative control shRNA HEK293 (1–21) cells, growth slowed down at approximately four days post serum starvation, and at seven days post serum withdrawal cell growth had almost completely stopped (Fig. 6A). However this was not observed with two different clonal derivative RanBPM shRNA cells (1–2 and 1–7). These cells continued to grow in the absence of serum, even at seven days post serum withdrawal. These results indicate that down-regulation of RanBPM expression promotes loss of growth factor dependence. In addition, as previous studies have demonstrated a role for the ERK pathway in promoting cell migration [34], [35], we tested the migratory properties of RanBPM and control shRNA HEK293 cells in a wound-healing assay, which evaluates the ability of cells to move over a cell-free zone created by scraping the middle of the plate with a pipette tip. This assay revealed a significant increase in cell motility of RanBPM shRNA cells, which displayed a 1.7 fold increase in wound closure compared to control cells (Fig. 6B and 6C). Altogether, these results indicate that loss of RanBPM expression leads to cell signaling alterations that promote aberrant cell proliferation and cell migration.

## Discussion

This study reveals an important role for RanBPM in repressing ERK activation and signaling. Expanding on our previous findings which showed elevated expression of the anti-apoptotic factor Bcl-2 in RanBPM down-regulated cells, we demonstrate here that Bcl-2 over-expression in these cells is mediated by increased ERK activation that is specifically triggered by the loss of RanBPM expression. We show that the inhibition of ERK signaling by RanBPM is achieved through a regulation of c-Raf protein levels and that RanBPM associates with c-Raf *in vivo*. Finally, we determine that loss of RanBPM expression confers increased cell growth and cell migration, properties known to be induced by increased ERK signaling.

We show here that the regulation of expression of Bcl-2 by RanBPM that we previously documented [21] is a direct consequence of a regulation of the ERK pathway by RanBPM. Our investigation of a potential effect of RanBPM on the ERK pathway was prompted by the observation that, in addition to Bcl-2, RanBPM also modulated the expression of another anti-apoptotic factor, Bcl-X_L_, and that these regulations occurred both at the transcriptional and at the post-translational level. The ERK pathway has previously been shown to regulate Bcl-2 and Bcl-X_L_ both transcriptionally and post-transcriptionally [4], [5], [27]. In our studies, we have shown that both transient and stable down-regulation of RanBPM activated ERK phosphorylation, and we have confirmed that this effect was specific to RanBPM, as restoration of RanBPM expression reversed this effect. Further, we have substantiated that RanBPM down-regulation promotes ERK activation in three different cell lines, confirming that the regulation of ERK by RanBPM is not cell-type specific. Moreover, we show that inhibiting ERK in RanBPM down-regulated cells reduces Bcl-2 expression, confirming that ERK signaling is directly responsible for Bcl-2 expression in these cells. Altogether, these experiments demonstrate that RanBPM expression has an inhibitory effect on ERK phosphorylation and signaling.

Previous studies have implicated RanBPM in the regulation of the ERK pathway, however there have been conflicting reports of the outcome of RanBPM expression on ERK activation. In a first study, RanBPM was shown to stimulate the ERK pathway through an interaction with the RTK MET [12]. While our results are in contradiction with this report, it should be noted that this previous study employed an experimental approach that involved over-expression of a GFP-RanBPM fusion construct. Whether the addition of a bulky fluorescent tag at the N-terminus of RanBPM interferes with RanBPM function is unknown, but since RanBPM has been shown to dimerize (or even multimerize) through the Lis1 homology (LisH) domain [36], it is possible that the GFP-RanBPM fusion protein may have had an adverse effect on endogenous RanBPM function. Another study presented evidence that the N-terminal region of RanBPM interacted with the neural cell adhesion molecule L1 and inhibited ERK activation induced by L1 [25]. However, this study relied on over-expression of a truncated form of RanBPM, and the effect of the truncation on RanBPM function was not investigated [25]. In contrast to these reports, a third study reported an inhibitory role of RanBPM on ERK signaling activated by a constitutively active Raf-BXB [24]. This study also described an interaction between RanBPM and the catalytic domain of c-Raf using a yeast two-hybrid assay. Consistent with these findings, we show here that RanBPM associates with c-Raf C-terminal region using a GST-pull-down assay in mammalian cells. Further, we also show that RanBPM can form a complex with endogenous c-Raf.

Raf proteins are central to ERK signaling as they integrate upstream signals, and thus are the targets of complex regulations [35]. Intensive studies on c-Raf in particular have revealed that c-Raf undergoes a complex cycle of activation/deactivation that involves multiple interactions with regulators, phosphorylation and dephosphorylation events, and conformational changes [35]. Our data indicate that RanBPM modulates c-Raf expression through a regulation of c-Raf protein levels/stability. First, our RT-PCR analyses revealed that RanBPM down-regulation does not results in increased c-Raf mRNA levels, indicating that RanBPM does not affect c-Raf gene expression. In fact, RanBPM down-regulation resulted in a slight, albeit not significant decrease in c-Raf mRNA levels. A possible explanation may be that elevated c-Raf protein expression activates negative feedback loops that repress its transcription. Second, our results show that RanBPM is able to modulate the stability of ectopically expressed c-Raf, as the expression of both active c-Raf point mutant (Y/Y) and ΔN deletion mutant was strongly affected by RanBPM expression. Interestingly, the down-regulation of c-Raf by RanBPM seemed more pronounced with these transfected active forms of c-Raf than with the pool of endogenous c-Raf proteins which comprises active and inactive forms. This is consistent with the ability of RanBPM to form a complex with the c-Raf C-terminal kinase domain, and thus suggests that RanBPM preferentially targets active forms of c-Raf and functions to regulate c-Raf through a regulation of its active form.

An important part of c-Raf regulation is its association with chaperone proteins that ensure proper folding and prevent c-Raf degradation [32]. c-Raf protein folding and stability has been shown to be dependent on its association with the chaperone Hsp90, as disruption of Hsp90-c-Raf interaction results in a sharp decrease in c-Raf levels [33], [37], [38]. Phosphorylation of Ser^621^ through c-Raf autophosphorylation has also been implicated in promoting c-Raf stability [39]. We did not find a consistent change in Ser^621^ phosphorylation resulting from RanBPM expression (data not shown). However, we found that RanBPM down-regulation enhanced c-Raf-Hsp90 complex formation. This effect was found to be specifically due to down-regulation of RanBPM, as restoration of RanBPM expression reduced the association of Hsp90 with c-Raf. These findings suggest that RanBPM interaction with c-Raf disrupts the c-Raf-Hsp90 complex, leading to destabilization of c-Raf. For instance, RanBPM may function to destabilize c-Raf by competing with Hsp90 for binding to c-Raf. Such a mechanism was previously proposed to explain the negative regulation of c-Raf by Hsp70. Hsp70 was shown to compete with c-Raf for binding to Bag-1, a chaperone that stimulates c-Raf catalytic activity, thus preventing c-Raf activation of proliferation pathways [40], [41]. Alternatively, RanBPM may recruit a protein or protein complex to c-Raf that disrupts the c-Raf-Hsp90 complex. As well, a precedent exists for such a remodeling of Hsp90-chaperone complexes, which is mediated by the co-chaperone CHIP (carboxy terminus of Hsp70-interacting protein) [42]. CHIP was shown to bind Hsp90 substrates and mediate the transfer of client proteins to Hsp70, causing their dissociation from Hsp90 and promoting their proteasome-mediated degradation [37], [42], [43], [44]. Whether RanBPM is part of this complex or functions independently in regulating chaperone-dependent Hsp90 client proteins stability/degradation remains to be determined.

It is well established that deregulation of the ERK pathway leading to its constitutive activation is linked with many aspects of tumour development including cell growth, proliferation, differentiation and migration [4], [5], [6]. Our observation that RanBPM down-regulation promotes ERK activation suggests that loss of RanBPM function could promote cellular events leading to cellular transformation. Both cell proliferation and cell migration were found enhanced in HEK293 RanBPM shRNA cells suggesting that RanBPM expression is essential to regulate these two cellular functions. It should be noted that while the increased cell migration observed upon RanBPM down-regulation may be due to increased ERK activation, it could also result from MEK-independent functions of c-Raf, which has been shown to regulate cell motility through a direct regulation of the Rho effector Rok-alpha [34], [35], [45]. Previous reports have suggested a function for RanBPM in repressing oncogenic cellular events by promoting the activity of the tumour suppressor p73 and Mammalian Lethal Giant Larvae-1 (Mgl-1) [17], [46]. Our results not only confirm a tumour suppressor role for RanBPM, but go beyond these observations to show that altering RanBPM expression is in itself sufficient to disrupt regulatory mechanisms that control cell transformation and the establishment of oncogenic pathways. Interestingly, decreased RanBPM expression was previously reported in cancer cells from several tumour samples, suggesting that loss of RanBPM may be linked to tumour development [47]. To confirm a link between these observations, it will be important to determine whether RanBPM loss of expression in tumours correlates with the constitutive activation of the ERK pathway.

## Materials and Methods

### Plasmids expression constructs

pCMV-HA-RanBPM shRNA mutant construct (HA-RanBPM si-mt) was previously described [21]. pEGFP-C1 is from Clontech (Mountain View, CA, USA), and pCGN-ΔN-Oct-1 has been reported elsewhere [48]. The pCMV-3xFlag-Bcl-2 construct was a kind gift from Dr. Sean P. Cregan (University of Western Ontario, London, ON, Canada). The kinase-deficient ERK1 construct pCEP4-DN-ERK1 (DN-ERK1) [49] was a kind gift from Dr. Melanie H. Cobb (University of Texas, Southwestern Medical Centre, Dallas, TX, USA). The constitutively active H-Ras construct pSV-3xHA-RasV12 (RasV12) [50] was a kind gift from Dr. Arthur Gutierrez-Hartmann (University of Colorado Denver, Aurora, CO, USA). The constitutively active c-Raf constructs pEBG-GST-ΔN-c-Raf (GST-ΔN-c-Raf) and pCMV-Flag-c-Raf Y340D/Y341D (Flag-Y/Y-c-Raf) [31], [51] were a kind gift from Dr. Zhijun Luo (Boston University, Boston, MA, USA).

### siRNA (small interfering RNA) and shRNA (small hairpin RNA) constructs

Control siRNA and RanBPM siRNA were purchased from Ambion (Austin, TX, USA) and have been previously described [21]. pSuper-shRanBPM and pSuper-shControl have been reported in [21].

### Cell culture and treatments

Hela and HCT116 control shRNA and RanBPM shRNA stable cell lines were generated previously [21], and HEK293 control shRNA and RanBPM shRNA stable cell lines were obtained similarly by clonal selection of cells transfected with pSuper-shRanBPM or pSuper-shControl vectors. Hela, HCT116, and HEK293 cells were cultured in high glucose Dulbecco's modified Eagle's medium (DMEM) supplemented with 10% fetal bovine serum (FBS) at 37°C in 5% CO_2_. Control shRNA and RanBPM shRNA stable Hela and HCT116 cell lines were maintained in media supplemented with 0.35 mg/ml G418 (Geneticin, Bioshop Canada, Burlington, ON, Canada) whereas HEK293 clonal derivatives were maintained in 0.45 mg/ml G418. For serum starvation experiments, HCT116 cells were cultured in media containing 0.1% FBS and HEK293 cells were cultured in serum-free media. For the MEK1/2 inhibitor (U0126) experiments, RanBPM shRNA Hela cells were treated with 10 µM U0126 (Cell Signaling, Danvers, MA, USA) or DMSO alone (Sigma, Oakville, ON, Canada) for 24 h.

### Transfections assays

Plasmid transfections were carried out with ExGen 500™ (MBI Fermentas, Burlington, ON, Canada) according to the manufacturer's protocol. siRNA duplexes were transfected with siPORT NeoFX (Ambion) as previously described [21].

### Western Blot, co-immunoprecipitations and GST-pull down assays

Whole cell extracts were prepared as described [21]. For experiments involving c-Raf analysis, the whole cell extract buffer was supplemented with 0.5% Triton-X-100. For Western blot analysis, extracts were resolved by SDS-PAGE (between 8% and 12%). Gels were transferred on PVDF membranes and hybridized with the following antibodies: RanBPM 5 M (Bioacademia, Japan), β-actin (I-19, Santa Cruz, Santa Cruz, CA, USA), Bcl-2 (Cell Signaling), Bcl-X_L_ (Cell Signaling), HA (HA-7, Sigma), Flag (M2, Sigma), phospho-T202/Y204-ERK1/2 (Cell Signaling), ERK1/2 (Cell Signaling), phospho-S217/221-MEK1/2 (Cell Signaling), MEK1/2 (Genscript, Piscataway, NJ, USA), c-Raf (clones C-12 and E-10, Santa Cruz), Hsp90 α/β (clone H-114, Santa Cruz), GST (GE Health Care Life Sciences, Baie d'Urfe, QC, Canada), γ-tubulin (a kind gift from Dr. David Litchfield, University of Western Ontario, London, ON, Canada). The blots were developed using the Western Lightning® Enhanced Chemiluminescence Reagent (Perkin Elmer, Waltham, MA, USA).

For co-immunoprecipitation analyses of RanBPM and c-Raf, 1.5 mg of extracts were adjusted to 0.25% Triton X-100, 0.25% NP-40 and 100 mM KCl, immunoprecipitations were carried out for 2 h at 4°C with the indicated antibodies, and immunoprecipitates were isolated with Dynabeads® protein G (Invitrogen, Life Technologies, Burlington, ON, Canada). GST pull-down assays were performed overnight in the same conditions with glutathione beads (Sigma). For co-immunoprecipitations of c-Raf and Hsp90, extracts were adjusted to 0.2% NP-40, 0.04% Triton X-100, and 100 mM KCl. The amount of extracts were adjusted to obtain similar amount of c-Raf immunoprecipitates from control and RanBPM shRNA cells, and immunoprecipitations were carried out overnight at 4°C with c-Raf (E-10) antibody and immunoprecipitates were isolated using Dynabeads®.

### Quantitative reverse-transcriptase PCR

Total RNA was collected from Hela control shRNA, RanBPM shRNA, and RanBPM shRNA re-expressing RanBPM si-mt, cells using the Qiagen RNeasy RNA Extraction kit (Qiagen, Mississauga, ON, Canada). cDNA was prepared from 2.5 µg of total RNA using the SuperScriptII Reverse Transcriptase kit (Invitrogen, Life Technologies). For gene expression analyses of Bcl-2 and Bcl-X_L_, 10 ng cDNA was incubated with control RNA polymerase II primers (Pol II) (FW: 5′ TTGCCTGTGGCTTGATGCG 3′ RV: 5′ TTTGTTCTTCCCGAGGATCAGC 3′); and 50 ng cDNA was incubated with either Bcl-2-specific primers (FW: 5′ TTGTTGTTGTTCAAACGGGA 3′ RV: 5′ ACAAAACCCCACAGCAAAAG 3′) or Bcl-X_L_-specific primers (FW: 5′ GTAAACTGGGGTCGCATTGT 3′ RV: 5′ CAGGTAAGTGGCCATCCAAG 3′). For c-Raf analysis, 10 ng cDNA was incubated with either control GAPDH primers (FW: 5′ GTAGCTCAGGCCTCAAGACCTTGG 3′ RV: 5′ TGCGGGCTCAATTTATAGAAACCG 3′) or c-Raf primers (FW: 5′ TTAATCGCGGGCGCTTGGGC 3′ RV: 5′ CCAGCTGACCCTTTTCGGGGC 3′). Quantitative real-time PCR analysis was performed using SYBR green (Bio-Rad, Missisauga, ON, Canada) and the Bio-Rad MyiQ single-colour real-time PCR detection system. Relative quantification of gene expression was determined by the ΔΔC(t) method, with Bcl-2, Bcl-X_L_ and c-Raf C(t) values normalized to that of the controls.

### Cell growth and cell migration assays

To assess cellular growth rates, control shRNA and RanBPM shRNA HEK293 cells were seeded in triplicate in 6-well dishes, and 24 h post-plating cells were placed in serum-free media. At each timepoint cells were trypsinized, counted using a hemocytometer, and the mean number of cells was determined. Percent growth was obtained by dividing the number of cells at each time point by the number of cells at day 1.

For cell migration assays, control and RanBPM shRNA HEK293 cells were grown to 100% confluence on 24-well dishes. Cell monolayers were incubated in the presence of 2 mM hydroxyurea (Sigma) for 24 h to prevent cell proliferation, after which cells were scratched using a sterile 200 µl pipette tip, washed, and maintained in DMEM supplemented with 2 mM hydroxyurea. Wound closure was assessed at 0 h and 24 h using a fluorescent microscope (IX70, Olympus), and images were captured using a charge-coupled device camera (Q-imaging). Percent migration was determined by measuring the wound width at each time point using ImageJ software.

### Statistical analyses

Statistical differences between groups was analyzed using a student's *t-*test and one-way analysis of variance (ANOVA) using GraphPad (GraphPad Software Inc., La Jolla, CA, USA). Results were considered significant when *P<0.05*.
